# T Cell and Antibody Response Following Double Dose of BNT162b2 mRNA Vaccine in SARS-CoV-2 Naïve Heart Transplant Recipients

**DOI:** 10.3390/diagnostics12092148

**Published:** 2022-09-03

**Authors:** Leen Delrue, Annelies Muylaert, Ann Beernaert, Imke De Pelsmaeker, Elly Boel, Ana Moya, Sofie Verstreken, Riet Dierckx, Ward Heggermont, Jozef Bartunek, Marc Vanderheyden

**Affiliations:** Cardiovascular Center, OLV Hospital, 9300 Aalst, Belgium

**Keywords:** SARS-CoV-2, vaccination, T-cell, antibody, heart transplantation

## Abstract

**Introduction:** Preliminary studies have suggested a low post-vaccination antibody response against severe acute respiratory syndrome coronavirus 2 (SARS-CoV-2) in heart transplant(HTx)recipients. Although many studies have focused on the role of antibodies in vaccine-induced protection against SARS-CoV-2, the role of T cell immunity is less well characterized. To date, data regarding seroconversion and T cell response after mRNA SARS-CoV-2 vaccination in patients undergoing HTx are scarce. Therefore, the present study aimed to assess the specific memory humoral and cellular responses after two doses of the BNT162b2 vaccine in HTx recipients. **Methods:** Blood was drawn from heart transplant (HTx) recipients at two pre-specified time points after the first and second vaccine doses to measure both the anti-SARS-CoV-2 antibody response against the spike protein and the SARS-CoV-2-reactive T cell response. **Results:** Our study included 34 SARS-CoV-2 naïve HTx recipients (mean age, 61 ± 11 years). The mean time from transplantation to the first vaccine dose is 10 ± 10 years. Subgroup analysis (n = 21) demonstrated that after the first vaccine dose, only 14% had antibodies and 19% had a SARS-CoV-2-reactive T-cell response, which increased to 41% and 53%, respectively, after the second dose. Interestingly, 20% of patients with no antibodies after the second dose still had a positive SARS-CoV-2-reactive T cell response. The percentage of patients with positive S-IgG antibody titers was significantly higher 5 years after transplantation (18% 0–5 years post-TX vs. 65% 5 years post-TX, *p* = 0.013). Similarly, 5 years after heart transplantation, the percentage of patients with a T cell response was significantly higher (35% 0–5 years post-TX vs. 71% 5 years post-TX, *p* = 0.030). **Conclusions:** In SARS-CoV-2 naïve HTx recipients, post-vaccination antibody titers but also SARS-CoV-2 specific T cell response are low. Therefore, the protection from SARS-CoV-2 that is generally attributed to vaccination should be regarded with caution in HTx recipients.

## 1. Introduction

Heart transplant recipients suffering from SARS-CoV-2 have an established case fatality of 25% [1]. Fortunately, vaccines to prevent coronavirus disease 2019 (COVID-19) have been shown to generate specific immune responses to viral antigens and neutralizing antibodies and reduce the risk and severity of symptomatic disease [2]. However, neither solid organ transplant nor immunocompromised patients were included in the phase 3 clinical trials of the mRNA vaccines. Despite the lack of information on safety and immunogenicity of these vaccines, both the European Society for Organ Transplantation and the American Society for Transplantation recommend the vaccination of solid organ transplant recipients, considering that the potential benefits of the vaccine likely outweigh its risks [3].

However, the efficacy, safety, and durability of SARS-CoV-2 mRNA vaccines in the heart transplant (HTx) population remains to be established. A recent study including 436 solid organ recipients reported no serious adverse events but an impaired immune response to the first dose of a mRNA vaccine (BNT162b2, Pfizer-BioNTech, or mRNA-1273, Moderna) [4] with anti-Spike IgG antibodies in only 14% of the Htx patients. Furthermore, Itzhaki et al. demonstrated that following the two-dose SARS-CoV-2 vaccine, no more than half of the HTx recipients generated anti-Spike -IgG antibodies [5].

Although many studies have focused on the antibody response and the role of antibodies in vaccine-induced protection against SARS-CoV-2, the details of T cell induction following vaccination remain incompletely understood. In healthy individuals T cells, particularly CD4^+^ cells, are primed by the vaccine and are detectable as early as 10–12 days after the first dose together with spike-specific antibodies, whereas neutralizing antibodies first appear after boost. Furthermore, longitudinal antigen-specific T cell analyses corroborate this observation by demonstrating a rapid vaccination-induced near-maximal antigen-specific CD4^+^ T cell response after the first vaccine dose, together with a more gradual and more variable CD8^+^ T cell response after the first and second dose [6] These observations point towards a key role of vaccine-induced T cells in early protection after prime vaccination [7] when neutralizing antibodies are low or non-existing.

To date, no data exist about seroconversion and T cell response or kinetics after mRNA SARS-CoV-2 vaccination in HTx patients. Therefore, the present study aimed to assess the specific memory humoral and cellular responses after two standard doses of the BNT162b2 vaccine in SARS-CoV-2 naïve HTx recipients.

## 2. Material and Methods

### 2.1. Studypopulation

This was a prospective single-center study conducted at the Cardiovascular Center, OLV Hospital, Aalst, Belgium. HTx recipients who received a two-dose SARS-CoV-2 mRNA vaccine (BNT162b2, Pfizer-BioNTech, Mainz, Germany) at a dose of 30 microgram each between March and September 2021 were included. Major exclusion criteria were HTx within the previous 30 days, patient’s refusal to get a two-dose vaccine schedule or to participate in the study, and a known prior SARS-CoV-2 infection (documented by nasopharyngeal swab RT-PCR testing).

Clinical and pharmacological immune-suppressive data were extracted from the patients’ electronic health records. All patients received standard immunosuppressive therapy with oral tacrolimus or cyclosporine, mycophenolate mofetil or azathioprine, and methylprednisolone. The study was approved by the institutional review board and patient approval was obtained.

### 2.2. Sample Processing

Venous blood for the assessment of the antibodies and detection of SARS-CoV-2-reactive T cells was collected 28 ± 13 days after the first (n = 21 patients) and 78 ± 27 days after the second vaccine dose (n = 34 patients) into sodium heparin and EDTA tubes by standard phlebotomy. Blood tubes were centrifuged at 3000 bpm for 15 min to separate plasma. Heparin and EDTA plasma were stored at −80 °C for antibody analysis.

### 2.3. Antibody Response

The Elecsys^®^ Anti-SARS-CoV-2 S immunoassay was used for the quantitative determination of antibodies (including IgG) to the SARS-CoV-2 spike (S) protein receptor binding domain (RBD) according to the manufacturer’s instructions (Roche Diagnostics International Ltd., Rotkreuz, Switzerland). The signal yield increases with the antibody titer and values ≥ 0.8 U/mL are interpreted as reactive [5].

### 2.4. T-Cell Response

Antigen-reactive T cells were identified and characterized by analyzing their effector functions [7,8], such as upregulation of activation markers and cytokine production. Here, the SARS-CoV-2 T Cell Analysis Kit (PBMC) (Miltenyi Biotec, Bergisch Gladbach, Germany) was used to efficiently assess the SARS-CoV-2-reactive T cell response based on a sensitive and precise multiparameter flow cytometry assay. This kit contains the SARS-CoV-2 PepTivator^®^ Peptide Pool of choice, antibodies for the identification of CD4^+^ and CD8^+^ T cells, for the exclusion of monocytes and B cells, as well as for the staining of activation markers and cytokines. Furthermore, a positive control (CytoStim), a live/dead marker (Viobility 405/452 Fixable Dye), and Brefeldin A and reagents (Inside Fix and Inside Perm) for the fixation and permeabilization of cells after stimulation are included.

**Figure 1:**
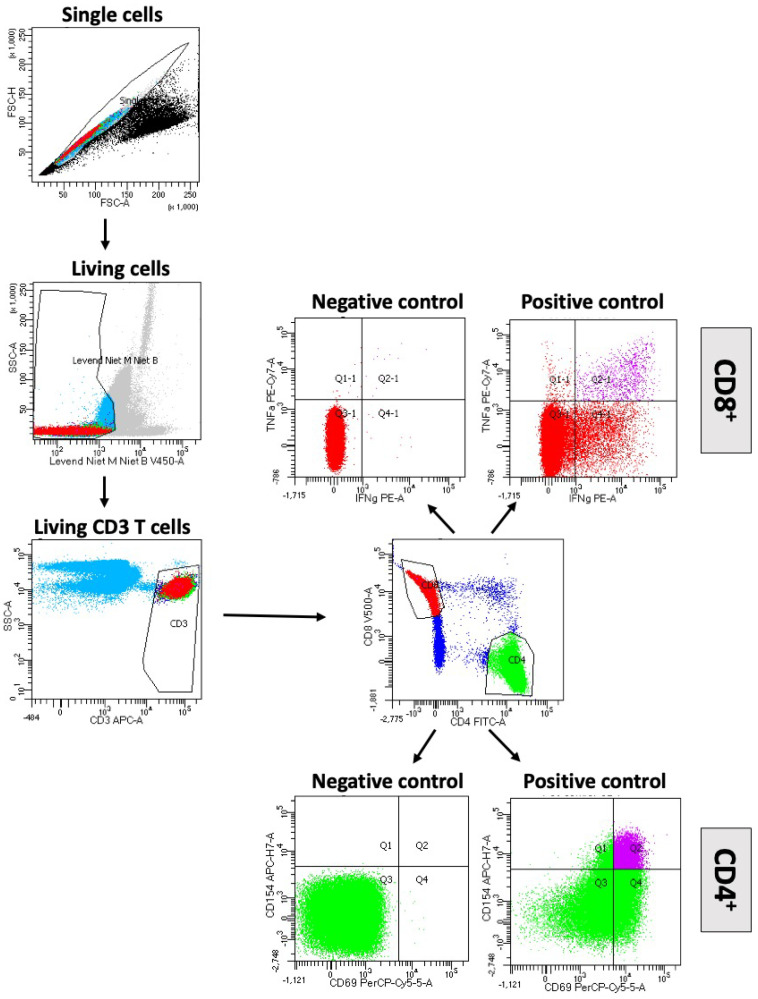
Induction of SARS-CoV-2 reactive T cells by M-, N- and S-protein overlapping peptide pools. Gating strategy for flow cytometry analyses of CD4^+^ and CD8^+^ T cells. Doublets, debris, and dead cells, as well as CD14^+^ and CD20^+^ cells were excluded. After identification of living CD3 cells CD4^+^ and CD8^+^ T cells are selected; CD4^+^ cells express CD154 and CD69 (Q2 Quadrant), CD8^+^ T cells TNF-α and IFN-γ (Q2 quadrant).

Freshly prepared human peripheral blood mononuclear cells (PBMCs) were incubated for a total of 6 h with a mix of the SARS-CoV-2 PepTivators peptide pool or left unstimulated (negative control). Peptide pools covering the sequences of the membrane (M) glycoprotein, nucleocapsid (N) phosphoprotein, and spike glycoprotein (S) were used. Polyclonal anti-CD3/CD28 T cell stimulation was used as a positive control. After 2 h of stimulation, Brefeldin A was added. Cells were then stained with the live/dead marker Viobility 405/452 Fixable Dye, fixed, and permeabilized. Afterwards, the cells were analyzed using a BD FACSCanto™ II analyzer (BD Biosciences). Doublets, debris, and dead cells, as well as CD14^+^ and CD20^+^ cells, were excluded. After pregating on CD3, as well as on CD4 and CD8, respectively, activation marker and cytokine expression were assessed, e.g., CD154 and CD69 for CD4^+^ T cells and TNF-α and IFN-γ for CD8^+^ T cells (Figure 1).

### 2.5. Statistical Analysis

Continuous variables with a normal distribution are presented as mean ± standard deviation and non-normally distributed variables are presented as median (interquartile range). Categorical variables were presented as percentages. *t*-tests or Mann–Whitney U tests were used according to the distribution of the variables for between-group comparisons. Statistical significance was set at *p* < 0.05. All analyses were performed using IBM SPSS Statistics version 25 software (IBM Corp., Armonk, NY, USA), Prism 7.0, (GraphPad Software Inc., San Diego, CA, USA), or calculated in R environment (R Foundation for Statistical Computing, Vienna, Austria) software version 3.4.1.0.

## 3. Results

### 3.1. Baseline Characteristics

The baseline characteristics of the HTx patients included in this study are summarized in Table 1. The mean time from HTx to the first vaccination was 10 ± 10 years. None of the patients experienced major adverse clinical events after vaccination, no episodes of rejection were detected. Two patients developed fever after the second vaccine. Blood samples were collected from 21 patients after the first and second vaccine doses. In the 13 other patients, blood was drawn only after the second dose.

### 3.2. Humoral and Cellular Immune Response after SARS-CoV-2 mRNA Vaccine

**Figure 2:**
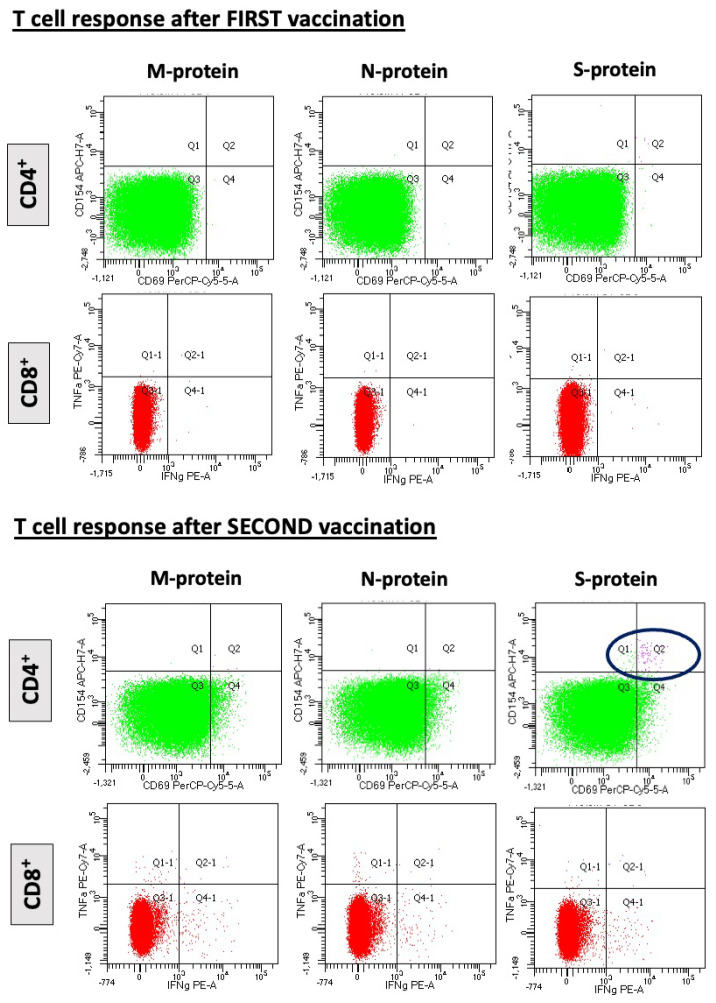
Representative example of a patient with SARS-CoV-2-reactive CD4 cells after stimulation with M-, N-, and S-protein overlapping peptide pools. Only after the second dose of BNT162b2 mRNA vaccination a CD4 cell response is observed (lower panel, blue circle). No CD8 cell response is noted after the first and second vaccine.

After the first vaccination, 14% of the patients were found to have induced S-IgG antibodies, which increased significantly to 41% after the second dose (*p* = 0.023). The percentage of HTx patients with functional T cell response increased from 19 to 53% (*p* = 0.015). Of note, 1 of 18 patients (5.6%) who did not produce antibodies after the first vaccine had reactive T cells. After the second dose, this percentage increased further, with 20% of those with no seroconversion showing a positive T cell response. Figure 2 demonstrates a representative example of a HTx patient with SARS-CoV-2-reactive CD4^+^ T cells after stimulation with M-, N-, and S-protein overlapping peptide pools.

In line with the results presented by the manufacturer, the overall response upon stimulation with SARS-CoV-2 PepTivator Peptide Pools was stronger for CD4^+^ T cells than for CD8^+^ T cells, which were only detected in 2 patients after the second vaccine.

To date, 12 patients who did not show an antibody response after the second dose were analyzed after receiving a third booster dose. In this cohort, seropositive S-IgG antibodies were detected in 6 out of 12 patients, while SARS-CoV-2 reactive CD4^+^ T cells were detected in 9 patients (T cell response could not be performed in 2 patients). Only one patient had neither an antibody or a functional T cell response.

### 3.3. Responders vs. Non-Responders

No difference in sex was noted between patients with positive S-IgG antibodies or functional T cell response vs. those without. The immunosuppressive regimen used was similar in both responders and non-responders (Table 1); tough responders were characterized by significantly lower tacrolimus trough levels (8.9 ± 1.5 vs. 11.9 ± 2.0 ng/mL, *p* < 0.001), compared to non-responders.

**Figure 3:**
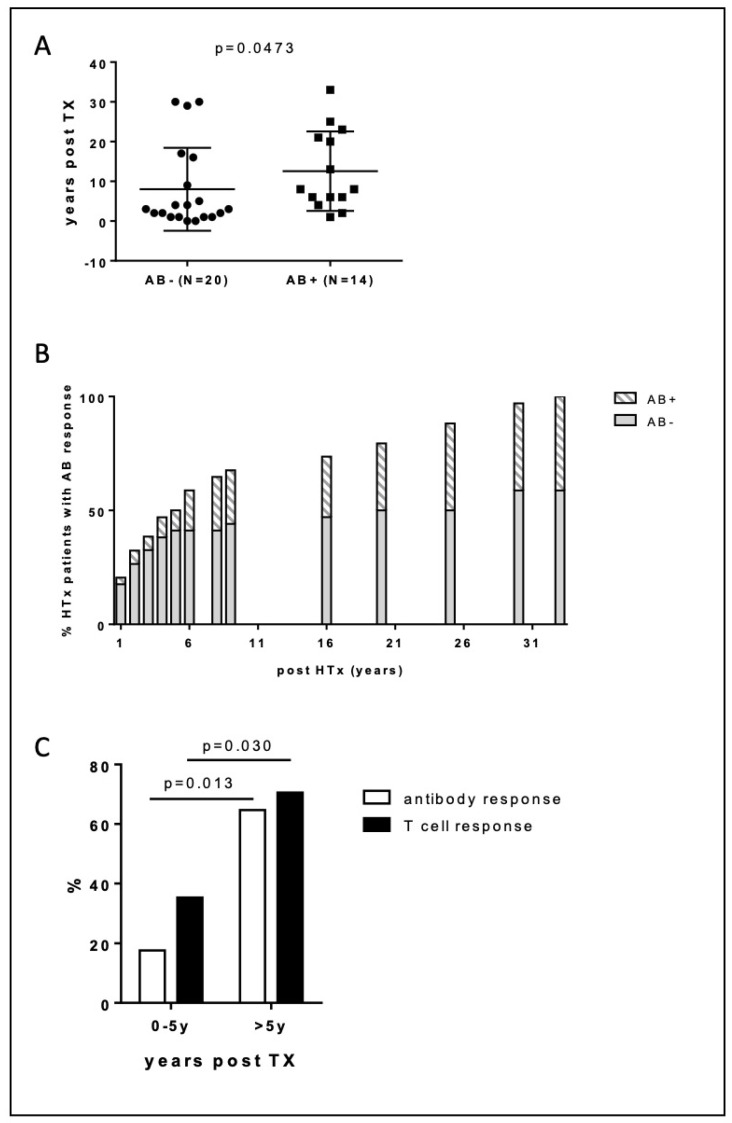
Antibody response according to years post transplantation. (**A**) Years post-transplantation in patients with and without antibody response after a full two-dose vaccination schedule. (**B**) Cumulative incidence of seroconversion after a full two-dose vaccination schedule according to years post-HTx. (**C**) Seropositive induction rates of SARS-CoV-2-specific T cell (black bar) and SARS-CoV-2 S-IgG (white bar) response after full two-dose vaccination schedule according to years post transplantation.

Furthermore, the antibody and cellular response was related to graft age with those without antibody or T cell response being more recently transplanted (AB^−^/CD4^+^ neg vs AB^+^/CD4^+^ pos: 13 ± 10 years vs. 8 ± 10 years, *p* = 0.0473) (Figure 3A). The percentage of patients with positive S-IgG antibody titers was significantly higher 5 years after transplantation (65% > 5 years post-HTx vs. 18% 0–5 years post-HTx, *p* = 0.013) (Figure 3B). Similarly, in those transplanted > 5 years, the percentage of patients with a positive T cell response was significantly higher (35% 0–5 years post-TX vs. 71% > 5 years post-HTx, *p* = 0.030) (Figure 3C). Interestingly, this was associated with significantly lower tacrolimus (9.0 ± 1.4 vs. 12.2 ± 1.9 ng/mL, *p* < 0.001) and cyclosporine trough levels (80 ± 11 vs. 121 ± 30 mg/dL, *p* < 0.001) in those transplanted > 5 years vs. those transplanted < 5 years).

While no difference was observed in age between non-responders and responders (63 ± 10 vs. 59 ± 11 years; *p* = ns), older HTx patients were more at risk of low immunogenicity (25% antibody response in patients >65 years vs. 52% in younger patients).

## 4. Discussion

Our results demonstrate that in SARS-CoV-2 naïve HTx patients, mRNA SARS-CoV-2 vaccines are safe with no major adverse effects and an absence of rejection following the vaccine administration. After a vaccination scheme of two doses of BNT162b2, not only the antibody but also the cellular response is impaired in COVID-19 naïve HTx patients, as evidenced by the low seroconversion rate of 41% and immune cellular response of 53%. This implies that, in this population, the generally achieved protection from SARS-CoV-2 attributed to vaccination is suboptimal and should be considered with caution. As both the anti-spike antibody and T cell response increase significantly after the second dose, future studies that evaluate the effects of a supplementary booster dose of BNT162b2 on antibody and specific T cell responses are warranted.

With an overall 20% mortality rate, solid organ transplant recipients have worse outcomes after SARS-CoV-2 infection [1]. Even when infected by the less lethal SARS-CoV-2 Omicron variant, hospital admission rate is higher and the duration of symptoms is more prolonged in HTx patients, compared to non-immunocompromised individuals [9]. Although a lot of effort and hope has been put on vaccines, as a strategy to protect this group of patients, the large randomized clinical trials excluded immunocompromised patients in their design. To date, only a few non-randomized observational studies have demonstrated the safety of these vaccines in this population. Our data corroborate previous findings and demonstrated that in heart transplant recipients, the vaccine is safe as evidenced by the low occurrence of minor adverse events and the absence of episodes of rejection in the immediate follow-up [3,5].

As compared to healthy controls, we observed lower antibody titers after the first and second doses, indicating poorer humoral response in heart transplant recipients. This poor serological response to vaccines in the solid organ transplant population is in line with a previous study [4], which demonstrated a low anti-S immunogenicity in a cohort of solid-organ transplant recipients, 15% of whom were HTx recipients. In an observational study, Itzhaki et al. reported that 49% of HTx recipients induced S-IgG antibodies in response to a two-dose vaccine schedule and that 36% of those who were non-responders to the first vaccine dose, became S-IgG seropositive after the second vaccine dose [5]. With 14% of the patients developing S-IgG antibodies after the first vaccination and 41% after the second dose, our study corroborates this observation.

Apart from humoral, T cell mediated immunity also plays an important role in neutralizing the virus following SARS-CoV-2 infection [8]. Of note, patients with inherited or treatment-induced B-cell deficiencies who fail to develop neutralizing antibodies recover from SARS-CoV-2 infection [10]. Similarly, in patients with hematological malignancies and SARS-CoV-2 infection, CD8^+^ T cells compensate for the lack of humoral immunity and are associated with an improved outcome [11,12].

The T cell response to mRNA vaccination is less well characterized. Initial reports indicate that T cells, particularly CD4^+^ cells, are primed by the vaccine [13,14]. In heart transplant recipients we confirmed this specific T cell-mediated immunity. However, in up to 47% of patients, no specific T cell response was observed after the second dose with the BNT162b2 vaccine highlighting that, like the humoral, the T cell-mediated immunity is impaired. A similar abnormal functional T cell response to SARS-CoV-2 with a lower count and impaired specific CD4^+^ cells has also been described after two doses of the BNT162b2 mRNA vaccine in older people and has been associated with frailty and age [15,16]. Using flow cytometry, it was shown that T cell subsets, which play a major role in the orchestration of the whole adaptive immune response, such as IFNg^+^ and triple^+^CD4^+^, were significantly lower in COVID-19-naive older participants [16].

The mechanism responsible for this impaired immunologic response remains undetermined. In solid organ transplant recipients’ older age, the presence of diabetes mellitus, the use of MPA or mycophenolate, high-dose corticosteroids, and triple immunosuppression therapy [17] have been associated with a negative antibody response.

Unfortunately, in our study, we were unable to discover a potential role for mycophenolate in the reduced humoral and T cell response, as most of our patients were on an anti-metabolite-based immunosuppression regimen. Interestingly, we noticed a better response with a higher seroconversion rate and T cell response after the mRNA SARS-CoV-2 vaccine in older grafts, compared to younger ones. Although speculative, the less intensive immunosuppressive regimen with lower trough levels of cyclosporine and tacrolimus late after heart transplantation may account for this observation. Moreover, the presence of higher tacrolimus trough levels in those with weak response points in this direction and corroborates previous observations, indicating an association between therapy with tacrolimus and weak humoral response after SARS-CoV-2 vaccine in solid organ transplant recipients [17].

### Limitations

This study has some limitations. First, this study was limited by its small sample size and single-center design. Second, although a baseline serological assessment was not performed, a prior SARS-CoV-2 infection could be ruled out, as these patients were followed up by the medical staff with a low threshold for SARS-CoV-2 screening. Third, we assessed the S-IgG antibody titer response to vaccines and not the SARS-CoV-2 neutralizing antibody titers. Nevertheless, S-IgG antibodies were found to correlate with the geometric mean titer of neutralizing antibodies, and thus they represent a surrogate for an adequate immune response [8]. As there are no precisely defined antibodies or T cell correlates of protection against SARS-CoV-2, we cannot be certain of the degree of clinical significance of the differences we report. Finally, this study addresses the occurrence and kinetics of vaccine-induced antibody and T cell production in SARS-CoV-2-naïve heart transplant recipients. Therefore, further studies exploring the differentiation state of vaccine-induced CD4^+^ and CD8^+^ T cells are needed to unravel the immunologic response to the mRNA vaccine in this specific patient population.

## 5. Conclusions

Poor anti-spike antibody and T cell responses in heart transplant recipients after the first and second doses of SARS-CoV-2 mRNA vaccines suggest that these patients may remain at a higher risk for COVID-19. Given the preliminary results of this study, either the administration of an additive booster vaccine or lowering or temporarily discontinuing antiproliferative immunosuppression, as a strategy to increase response to SARS-CoV-2 vaccines, similar to what has been tested in kidney transplant recipients [17,18], might be promising and deserves to be explored in future research.

## Figures and Tables

**Table 1:** Baseline characteristics of the HTx patients subdivided according to their S-IgG immunogenicity (S-IgG pos vs. S-IgG neg) and T cell response (CD4 pos vs. CD4 neg) following a two-dose SARS-CoV-2 vaccination.

	All Patients (n = 34)	S-IgG Pos (n = 14)	S-IgG Neg (n = 20)	CD4 Pos (n = 18)	CD4 Neg (n = 16)
Age (years)	61 ± 11	59 ± 11	63 ± 10	60 ± 11	63 ± 11
Male/Female (%)	25 (74)/9 (26)	8 (57)/6 (43)	17 (85)/3 (15)	11 (61)/7 (39)	14 (88)/2 (13)
Time from HTx (years)	10 ± 10	13 ± 10	8 ± 10 *	11 ± 10	8 ± 11
Blood draw post second vaccination (days)	78 ± 27	76 ± 22	79 ± 30	83 ± 30	72 ± 22
Medication					
CNI					
Tacrolimus	23 (68)	8 (57%)	15 (75)	10 (56%)	12 (75%)
Ciclosporin	12 (35)	6 (43%)	6 (30%)	8 (44%)	3 (19%)
MMF	25 (74%)	9 (64%)	16 (80%)	12 (67%)	12 (75%)
CD4 positive	18 (53)	14 (100)	4 (20)	/	/
AB positive	14 (78)	/	/	14 (78)	0 (0)
Blood group					
A (%)	18 (53)	7 (50)	11 (55)	7 (39)	11 (69)
B (%)	2 (6)	1 (7.1)	1 (5)	1 (6)	1 (6)
O (%)	12 (35)	4 (28.6)	8 (40)	8 (44)	4 (25)
AB (%)	2 (6)	2 (14.3)	0 (0)	2 (11)	0 (0)
eGFR	49 ± 18	54 ± 20	46 ± 15	52 ± 19	47 ± 15

* indicates *p* < 0.05 S-IgG pos vs. S-IgG neg.
